# Perceptions and Impact of the 2017 Canadian Guideline for Opioid Therapy and Chronic Noncancer Pain: A Cross-Sectional Study of Canadian Physicians

**DOI:** 10.1155/2020/8380171

**Published:** 2020-02-17

**Authors:** Jason W. Busse, Joyce Douglas, Tara S. Chauhan, Bilal Kobeissi, Jeff Blackmer

**Affiliations:** ^1^Department of Anesthesia, McMaster University, Hamilton, ON, Canada; ^2^Michael G. DeGroote Institute for Pain Research and Care, McMaster University, Hamilton, ON, Canada; ^3^Department of Health Research Methods, Evidence and Impact, McMaster University, Hamilton, ON, Canada; ^4^Michael G. DeGroote Centre for Medicinal Cannabis Research, McMaster University, Hamilton, ON, Canada; ^5^Canadian Medical Association, Ottawa, ON, Canada

## Abstract

**Background:**

Physician adherence to guideline recommendations for the use of opioids to manage chronic pain is often limited.

**Objective:**

In February 2018, we administered a 28-item online survey to explore perceptions of the 2017 Canadian guideline for opioid therapy and chronic noncancer pain and if physicians had altered practices in response to recommendations.

**Results:**

We invited 34,322 Canadian physicians to complete our survey, and 1,128 responded for a response rate of 3%; 687 respondents indicated they prescribed opioids for noncancer pain and answered survey questions about the guideline and their practice. Almost all were aware of the guideline, 94% had read the document, and 89% endorsed the clarity as good or excellent. The majority (86%) felt the guideline was feasible to implement, but 66% highlighted resistance by patients, and 63% the lack of access to effective nonopioid treatment as barriers. Thirty-six percent of respondents mistakenly believed the guideline recommended mandatory tapering for patients using high-dose opioid therapy (≥90 mg morphine equivalent per day), and 58% felt they would benefit from support for opioid tapering. Seventy percent of respondents had changed practices to align with guideline recommendations, with 51% engaging some high-dose patients in opioid tapering and 43% reducing the number of new opioid starts.

**Conclusion:**

There was high awareness of the 2017 Canadian opioid guideline among respondents, and preliminary evidence that recommendations have changed practice to better align with the evidence. Ongoing education is required to avoid the misunderstanding that opioid tapering is mandatory, and research to identify effective strategies to manage chronic noncancer pain is urgently needed.

## 1. Introduction

Canada is the world's second largest per capita prescriber of opioids [1], and escalating rates of opioid overdoses and deaths [2], in conjunction with increased recognition of the limited effectiveness of opioids for chronic noncancer pain [3], have prompted concerns. In May 2017, the updated Canadian guideline for opioids for chronic noncancer pain was published in the Canadian Medical Association Journal (CMAJ) to promote evidence-based prescribing [4]. However, prior studies have found limited adherence by physicians to guideline recommendations for the use of opioids to manage chronic pain [5, 6], explained in part by lack of awareness and familiarity with guidelines [7, 8].

The 2017 guidelines were supported by many Canadian medical organizations, including members of the Pan-Canadian Collaborative for Improved Opioid Prescribing (e.g., the Canadian Medical Association (CMA), the Association of Faculties of Medicine of Canada, the College of Family Physicians of Canada, the Federation of Medical Regulatory Authorities of Canada, and the Royal College of Physicians and Surgeons of Canada) [9]. To increase awareness, the Collaborative included a supplement in CMAJ in June 2017 which summarized the guideline [10]. In 2018, the CMA, in partnership with McMaster University, surveyed Canadian physicians to determine their perceptions of the 2017 Canadian opioid guideline, and whether they had made changes in their management of chronic noncancer pain patients as a result of the guideline's recommendations.

## 2. Materials and Methods

Through a partnership between McMaster University and the CMA and with the assistance of pain physicians, methodologists, and members of the Pan-Canadian Collaborative for Improved Opioid Prescribing, we developed a 28-item English and French-language questionnaire to examine awareness and perceptions of the 2017 Canadian opioid guideline among Canadian physicians who prescribe opioids for chronic noncancer pain. Respondents were also asked about their preferences for a traditional print format or an online version through MAGICapp (https://app.magicapp.org/app#/guidelines), an online platform that provides guidelines in an interactive, multilayered format. We further queried if respondents had made any changes to their practices in response to guideline recommendations. We tested the questionnaire with members of the collaborative, and changes were made to include questions regarding supports needed by physicians. We pretested the final questionnaire on a group of 3 physicians who prescribed opioids for chronic noncancer pain, who also commented on its clarity and comprehensiveness and on the time required to complete it (5–8 minutes). No further modifications were suggested by pretest participants ([Supplementary-material supplementary-material-1]). The Hamilton Integrated Research Ethics Board (HiREB) approved our survey for dissemination without ethics review.

The CMA distributed the questionnaire, on February 1, 2018, by emailing to a random sample of 34,322 of their members. Physicians were provided with a disclosure letter detailing the intent of the survey and explicit instructions that they could choose not to complete the survey or withdraw at any time. The survey was made available for one month, and three reminder emails were sent at one-week intervals during the study period. Survey respondents were anonymized with the use of a unique identifying number assigned to each participant. We generated frequencies for all collected data and reported categorical data as proportions.

## 3. Results

Of 34,322 physicians approached to complete our survey, 1,128 responded for a response rate of 3%. Six hundred and eighty-seven respondents, who indicated they prescribed opioids for noncancer pain, were eligible to complete specific questions about the guideline and their practice and chose to do so. Approximately half of the eligible respondents were under 55 years of age, 59% were male (394 of 668), and most were family physicians (68%; 463 of 678) practicing in Ontario (41%; 273 of 667). Forty-two percent (282 of 676) reported that >10% of their patients sought care for chronic noncancer pain, and 36% (214 of 596) prescribed opioids to >10% of their patients (Table 1).

### 3.1. Awareness of the 2017 Canadian Opioid Guideline

Almost all respondents were aware of the 2017 Canadian opioid guideline (92%; 630 of 686) and had read it thoroughly (45%; 280 of 626) or in part (50%; 310 of 626). Similar proportions had read the print (39%; 229 of 588) and online MAGICapp (43%; 250 of 588) versions; 19% (109 of 588) had read both. The large majority of respondents endorsed both print and MAGICapp formats as good or excellent (82% for both); however, twice as many endorsed the online MAGICapp version as excellent (18% vs. 9%).

Eighty-nine percent of respondents agreed the clarity of the guideline was excellent (17%; 93 of 539) or good (72%; 387 of 539). Some respondents (36%; 195 of 538) felt the 2017 Canadian opioid guideline had advantages over competing guidelines, whereas 49% (262 of 538) were unsure and 12% disagreed (63 of 538). Those who reported advantages most often highlighted its national scope (59%; 114 of195) and specific guidance (53%; 103 of 195). Sixty percent agreed the 2017 Canadian opioid guideline was evidence-based; 32% were unsure, and 8% disagreed. (Table 2) When asked if the guideline mandated tapering all patients prescribed high-dose opioids for chronic noncancer pain to <90 mg morphine equivalent per day (MED), 51% (269 of 529) disagreed, 36% (193 of 529) agreed, and 13% (67 of 529) were unsure.

### 3.2. Barriers and Facilitators of Guideline Implementation

Eight-six percent of respondents felt the guideline was either very feasible (15%; 85 of 553) or somewhat feasible (70%; 389 of 553) to implement. Of the 14% (79 of 553) that felt the guideline was not feasible to implement, the most common concerns were that recommendations were too restrictive regarding the use of opioids (60%; 47 of 79) or that recommendations were impractical (58%; 46 of 79).

Most respondents acknowledged resistance by patients (66%; 388 of 590), financial barriers to nonpharmacologic treatment (63%; 374 of 590), and lack of availability of nonpharmacologic treatment (63%; 374 of 590) as implementation challenges. Many respondents noted inadequate time to manage complex cases (46%; 273 of 590), lack of access to addiction management services (45%; 266 of 590), and that it was unrealistic for some of their high-opioid dose legacy patients to engage in tapering (41%; 243 of 590) (Table 3). When asked if they would benefit from support to help taper high-dose opioid patients, 58% (264 of 452) agreed; 16% (70 of 452) were uncertain, and 26% (118 of 452) disagreed. Eighty-two respondents did not reply as they reported no patients in their practice that were prescribed ≥90 mg MED.

Most respondents felt that increased coverage for chronic pain treatment options by insurers (60%; 353 of 590), and greater availability of chronic pain treatment services (55%; 323 of 590) would facilitate guideline implementation. Forty percent of respondents (235 of 590) indicated that billing incentives were required to encourage physicians to spend more time with their chronic noncancer pain patients. The most requested topics for continuing medical education (CME) among respondents were for assistance managing demanding, resistant, or nonadherent patients on chronic opioids (58%; 339 of 590) and instruction on nonopioid options for managing chronic noncancer pain (44%; 262 of 590) (Table 3). The most desirable formats for continuing medical education were small groups (31%; 160 of 509) and online courses or archived videos (23%; 117 of 509).

### 3.3. Changes in Practice due to the Guideline

The majority of respondents (57%; 318 of 558) agreed there were areas where their practice had differed from recommendations of the 2017 Canadian opioid guideline, and 79% said they had (70%) or intended (9%) to make changes to their practice because of guideline recommendations. Only 16% (96 of 590) reported they did not intend to make any changes to their practice. Specifically, in response to the guideline, 51% (301 of 590) of respondents had engaged some of their legacy patients in opioid tapering, and 43% had reduced the number of new starts of opioid therapy (254 of 590). Approximately a third (36%; 215 of 590) had reduced the number of opioid tablets they prescribed at one time, and 32% (191 of 590) had reduced the dose of opioids they prescribed for new starts. A small minority of physicians had either stopped prescribing opioids for chronic noncancer pain (8%; 46 of 590) or increased their prescribing of opioids for chronic pain (1%; 5 of 590) (Table 4).

## 4. Discussion

Almost all opioid-prescribing survey respondents were aware of the 2017 Canadian opioid guideline and endorsed its clarity and feasibility of implementation; the majority agreed the guideline was evidence-based. Resistance from patients and lack of access to effective nonpharmacologic therapy for chronic pain were the main reported barriers to implementation, and most respondents wanted assistance with tapering high-dose opioid patients. Only half of our respondents were confident that the guideline did not mandate tapering for patients prescribed high-dose opioid, and the majority had altered their practices as a result of the guideline, most often involving tapering legacy patients and reducing the number of new starts of opioid therapy for chronic noncancer pain patients.

### 4.1. Relevant Literature

The 2017 guideline was published with the MAGICapp online platform due to feedback that print versions were cumbersome to use in practice [11], and twice as many respondents rated the MAGICapp version vs. the print version as excellent. The primary reported barrier to guideline implementation was patient reluctance, and the most requested CME topic by respondents was management of difficult patients. Many physicians find patients with chronic noncancer pain challenging [12], and a 2017 qualitative study of family physicians in Ontario, Canada, found many did not feel equipped to navigate discussions with chronic pain patients with the result that some avoided prescribing opioids in order to avoid challenging discussions. Some physicians also reported blaming their College or guidelines as a way to escape conflict with patients when pursuing opioid tapering [13].

Physicians are well-trained in prescribing, but not always in deprescribing, and a number of our survey respondents highlighted this challenge; the third most requested CME topic was strategies to safely taper opioids. There is likely a dose-dependent increase in the risk of nonfatal and fatal opioid overdose [14, 15]. Moreover, low quality evidence suggests that opioid tapering is often successful and may improve patients' pain, function, and quality of life [16, 17]; however, all evidence relates to voluntary tapering. Mandatory opioid tapering is not recommended by the 2017 Canadian opioid guideline [4] and should not be pursued [18], in part because loss of prescription opioids, and resulting withdrawal symptoms may cause some patients to seek out illicit, more dangerous sources [19].

It is noteworthy that approximately a third of respondents to our survey felt the 2017 guideline endorsed mandatory opioid tapering among patients prescribed high-dose opioid therapy, and this may reflect confusion between weak and strong recommendations. Recommendation 9 of the guideline, which suggests tapering opioids to the lowest effective dose for patients prescribed ≥90 mg MED, potentially including discontinuation, rather than making no change in opioid therapy, is a weak recommendation. This means that while the majority of informed patients would choose the recommended course of action, an appreciable minority would not. With weak recommendations, clinicians should recognize that different choices will be appropriate for individual patients, and they should help patients arrive at decisions consistent with their values and preferences. The online version of the 2017 Canadian guideline includes patient decision aids for all weak recommendations (http://www.magicapp.org/public/guideline/8nyb0E).

On October 18, 2019, the College of Physicians and Surgeons of Alberta, HELP Alberta's Pain (a patient advocacy group; https://www.helpalbertaspain.com/), the Alberta Medical Association Section of Chronic Pain, and the Pain Society of Alberta signed an Agreement in Principle to support chronic pain patients prescribed long-term opioid therapy (https://www.painab.ca/uploads/1/0/8/0/108066059/aip.pdf). This document, which sites the 2017 Canadian Opioid Guideline for support, advises, in part, that no individual on long-term opioid therapy should be abruptly discontinued. Similar agreements in other provinces may be helpful for re-enforcing this message among prescribers.

Many respondents felt that greater access to effective chronic pain management was required; however, while nonopioid alternatives would be expected to reduce harms associated with long-term opioid use, the incremental benefit for chronic pain sufferers is uncertain. Pharmacologic therapies that have been directly compared with opioids show similar, small effects on pain and function [3]. Moreover, a study of 45,303 US adults with back and neck pain found that although total healthcare expenditures adjusted for inflation increased 65% from 1997 to 2005, from $52 to $86 billion/year, age- and sex-adjusted self-reported measures of mental health, physical functioning, work limitations, and social limitations were worse in 2005 than in 1997 [20]. Canada spends approximately $7.2 billion per year in direct healthcare for chronic noncancer pain [21]; however, from 2003 to 2008, the average funding for pain research in Canada was $17 million per year, which represented <1% of total funding from the Canadian Institutes of Health Research and <0.25% of total health research funding in Canada [22]. It may be that greater investment in research is needed to establish more effective strategies for chronic pain versus simply providing greater access to currently available therapies.

### 4.2. Strengths and Limitations

Strengths of our study include a comprehensive sampling of Canadian physicians who prescribe opioids for chronic noncancer pain. Moreover, our survey development and administration was consistent with best practices [23]. Our findings are limited by our low response rate (3%), which was similar to a prior survey of Canadian physicians' practices regarding opioid management of chronic noncancer pain (710 of 32,000; 2% response rate) [24], and greatly reduces the generalizability of our results.

## 5. Conclusions

There was very high awareness of the 2017 Canadian guideline for opioid therapy and chronic noncancer pain among respondents to our survey, and most physicians reported changing their practices to align with recommendations. Optimizing opioid-prescribing for chronic noncancer pain should reduce opioid-related harms, but confirmation from rigorously conducted observational studies is required. Further education appears needed to ensure appropriate interpretation of weak and strong guideline recommendations, and re-enforcement that forced opioid tapering is inappropriate. The Canadian opioid crisis has highlighted the urgent need for improved management of chronic pain; however, the lack of highly effective therapies suggests that further research is urgently needed versus simply expanding access to existing treatment options.

## Figures and Tables

**Table 1:** Demographic characteristics of survey responders.

Age (*n* = 674), *n* (%)	
<35	86 (13%)
35–44	114 (17%)
45–54	158 (23%)
55–64	204 (30%)
≥65	112 (17%)

Sex, (*n* = 668), *n* (%)	
Male	394 (59%)
Female	274 (41%)

Training, (*n* = 678), *n* (%)	
Family physician	463 (68%)
Specialist	215 (32%)

Province or territory of practice, (*n* = 667), (%)	
Ontario	273 (41%)
British Columbia	128 (19%)
Alberta	121 (18%)
Manitoba	36 (5%)
Quebec	29 (4%)
Saskatchewan	29 (4%)
Nova Scotia	29 (4%)
New Brunswick	10 (2%)
Other^*∗*^	12 (2%)

Practice setting, (*n* = 680), *n* (%)	
Urban/suburban	484 (71%)
Rural/geographically isolated	100 (15%)
Small town	96 (14%)

Proportion of patients seeking care for chronic noncancer pain, (*n* = 676), *n* (%)	
<6%	218 (32%)
6–10%	176 (26%)
11–20%	154 (23%)
21–50%	75 (11%)
51–75%	26 (4%)
>75%	27 (4%)

Proportion of chronic noncancer pain patients prescribed opioids (*n* = 596), *n* (%)	
<6%	253 (42%)
6–10%	129 (22%)
11–20%	78 (13%)
21–50%	84 (14%)
51–75%	27 (5%)
>75%	25 (4%)

^*∗*^Newfoundland (*n* = 5, 0.7%); Yukon Territory (*n* = 3, 0.4%); Prince Edward Island (*n* = 2, 0.3%); Northwest Territories (*n* = 2, 0.3%).

**Table 2:** Respondents impressions of the 2017 Canadian opioid guideline.

Awareness of the guideline (*n* = 686), *n* (%)	
Yes	630 (92%)
No	56 (8%)

Guideline version read (*n* = 588), *n* (%)	
Print version only	229 (39%)
Online MAGICapp version only	250 (43%)
Print and online MAGICapp versions	109 (18%)
Neither	

Format of the print version (*n* = 326), *n* (%)	
Excellent	29 (9%)
Good	239 (73%)
Unsure/do not recall	31 (10%)
Poor	24 (7%)
Very poor	3 (1%)

Format of the online MAGICapp version (*n* = 345), *n* (%)	
Excellent	63 (18%)
Good	219 (64%)
Unsure/do not recall	38 (11%)
Poor	23 (7%)
Very poor	2 (1%)

Advantages over competing opioid guidelines (*n* = 195), *n* (%)^1^	
National in scope	114 (59%)
More specific guidance	103 (53%)
Broadly endorsed	94 (48%)
Better evidence review process	83 (43%)

The 2017 guideline is evidence-based (*n* = 505), *n* (%)	
Strongly agree	65 (13%)
Agree	240 (48%)
Uncertain	160 (32%)
Disagree	36 (7%)
Strongly disagree	4 (1%)

^1^Percentages add up to >100% as respondents could endorse more than one option.

**Table 3:** Barriers and facilitators to implementation of the 2017 Canadian opioid guideline.

Implementation challenges (*n* = 590), *n* (%)^1^	
Reluctance by patients	388 (66%)
Financial barriers to nonpharmacologic treatment alternatives	374 (63%)
Lack of nonpharmacologic treatment alternatives	371 (63%)
Inadequate time to deal with complex cases	273 (46%)
Lack of access to addiction management services	266 (45%)
Unrealistic to taper some high-dose legacy patients to <90 mg MED	243 (41%)
Lack of access to specialists for support	218 (37%)
Financial barriers to addiction management services	189 (32%)
Need better training in chronic pain management	127 (22%)
Need more training in chronic pain management	118 (20%)
Need better training in addiction management	114 (19%)

Implementation facilitators (*n* = 590), *n* (%)^1^	
More chronic pain treatment options covered by insurance	353 (60%)
Greater availability of chronic pain treatment services	323 (55%)
Billing incentives to spend more time with chronic pain patients	235 (40%)
Greater availability of addiction treatment services	221 (38%)
Continuing medical education on opioid deprescribing	202 (34%)
Continuing medical education on nonopioid management of chronic pain	180 (31%)
Real-time access to a prescription monitoring database	153 (26%)
Access to treatment to support behavioral change	126 (21%)
Clinical decision support system integrated into EMRs	116 (20%)
Access to support, such as self-assessments, checklists, and algorithms	120 (20%)
Continuing medical education on opioid prescribing	109 (19%)
Mentorship programs in chronic noncancer pain and addiction	90 (15%)

Continuing medical education topics of interest (*n* = 590), *n* (%)^2^	
Managing demanding, resistant, or nonadherent patients on chronic opioids	339 (58%)
Nonopioid options for chronic noncancer pain management	262 (44%)
Strategies to safely taper opioids	209 (35%)
Initiating opioid substitution therapy	190 (32%)
Community programs to reduce opioid addiction and deaths	117 (20%)
Screening, initiating, and monitoring patients on opioid therapy	106 (18%)
Preventing and managing opioid overdoses	38 (6%)

^1^Percentages add up to >100% as respondents could endorse more than one option. ^2^Percentages add up to >100% as respondents could endorse up to 3 options. EMRs: electronic medical records.

**Table 4:** Changes in practice due to the 2017 Canadian opioid guideline.

New practice behaviors (*n* = 590), *n* (%)^1^	
Engaged some legacy patients in opioid tapering	301 (51%)
Reduced the number of new starts of opioids for chronic noncancer pain	254 (43%)
Reduced the number of pills dispensed at one time, for opioid prescriptions	215 (36%)
Reduced the dose of opioids for new starts	191 (32%)
Avoid prescribing opioids for chronic noncancer pain patients with an active substance use disorder	161 (27%)
Avoid prescribing opioids for chronic noncancer pain patients who also have an active psychiatric disorder, aside from substance use disorder	102 (17%)
Avoid prescribing opioids for specific patients with chronic noncancer pain based on criteria other than having an active substance use disorder or an active psychiatric disorder	96 (16%)
Avoid prescribing opioids for any chronic noncancer pain patients	46 (8%)
Prescribe opioids to more patients with chronic noncancer pain	5 (1%)

^1^Percentages add up to >100% as respondents could endorse more than one option.

## Data Availability

The study data used to support the findings of this study are included within the article.

## Supplementary Materials

Supplementary Materials Appendix: Opioid Guideline Survey.

## References

[1] International Narcotics Control Board (INCB) (2016). Narcotic drugs: estimated world requirements for 2016.

[2] Opioid-related harms and deaths in Canada. https://www.canada.ca/en/health-canada/services/substance-use/problematic-prescription-drug-use/opioids/data-surveillance-research/harms-deaths.html.

[3] Busse J. W., Wang L., Kamaleldin M. (2018). Opioids for chronic noncancer pain. *JAMA*.

[4] Busse J. W., Juurlink D. N., Buckley D. N., Guyatt G. H. (2017). The authors respond to “inconsistencies in the 2017 Canadian guideline for opioids for chronic noncancer pain”. *Canadian Medical Association Journal*.

[5] Khalid L., Liebschutz J. M., Xuan Z. (2015). Adherence to prescription opioid monitoring guidelines among residents and attending physicians in the primary care setting. *Pain Medicine*.

[6] Martinez V., Attal N., Vanzo B. (2014). Adherence of French GPs to Chronic Neuropathic Pain Clinical Guidelines: results of a Cross-Sectional, Randomized, “e” Case-Vignette Survey. *PLoS One*.

[7] Krebs E. E., Bergman A. A., Coffing J. M., Campbell S. R., Frankel R. M., Matthias M. S. (2014). Barriers to guideline-concordant opioid management in primary care-A qualitative study. *The Journal of Pain*.

[8] Cabana M. D., Rand C. S., Powe N. R. (1999). Why don’t physicians follow clinical practice guidelines?. *JAMA*.

[9] College of Family Physicians of Canada (July 2019). About the Pan Canadian collaborative. https://www.cfpc.ca/chronic-non-cancer-pain-management-opioid-resources-about/.

[10] Canadian Medical Association (CMA) (July 2017). 2017 Canadian opioid prescribing guideline. https://www.cma.ca/sites/default/files/2018-11/2139_cma_opioid_poster_en.pdf.

[11] Chang Y., Zhu K. L., Florez I. D. (2016). Attitudes toward the Canadian guideline for safe and effective use of opioids for chronic non-cancer pain: a qualitative study. *Journal of Opioid Management*.

[12] Sharpe M., Mayou R., Seagroatt V. (1994). Why do doctors find some patients difficult to help?. *QJM: An International Journal of Medicine*.

[13] Desveaux L., Saragosa M., Kithulegoda N., Ivers N. M. (2019). Understanding the behavioural determinants of opioid prescribing among family physicians: a qualitative study. *BMC Family Practice*.

[14] Dunn K. M., Saunders K. W., Rutter C. M. (2010). Opioid prescriptions for chronic pain and overdose. *Annals of Internal Medicine*.

[15] Kaplovitch E., Gomes T., Camacho X., Dhalla I. A., Mamdani M. M., Juurlink D. N. (2015). Sex differences in dose escalation and overdose death during chronic opioid therapy: a population-based cohort study. *PLoS One*.

[16] Frank J. W., Lovejoy T. I., Becker W. C. (2017). Patient outcomes in dose reduction or discontinuation of long-term opioid therapy. *Annals of Internal Medicine*.

[17] Darnall B. D., Ziadni M. S., Stieg R. L., Mackey I. G., Kao M.-C., Flood P. (2018). Patient-centered prescription opioid tapering in community outpatients with chronic pain. *JAMA Internal Medicine*.

[18] Darnall B. D., Juurlink D., Kerns R. D. (2019). International stakeholder community of pain experts and leaders call for an urgent action on forced opioid tapering. *Pain Medicine*.

[19] Busse J. W., Juurlink D., Guyatt G. H. (2016). Addressing the limitations of the CDC guideline for prescribing opioids for chronic noncancer pain. *Canadian Medical Association Journal*.

[20] Martin B. I., Deyo R. A., Mirza S. K. (2008). Expenditures and health status among adults with back and neck problems. *JAMA*.

[21] Hogan M.-E., Taddio A., Katz J., Shah V., Krahn M. (2016). Incremental health care costs for chronic pain in Ontario, Canada. *Pain*.

[22] Lynch M. E., Schopflocher D., Taenzer P., Sinclair C. (2009). Research funding for pain in Canada. *Pain Research and Management*.

[23] Burns K. E. A., Duffett M., Kho M. E. (2008). A guide for the design and conduct of self-administered surveys of clinicians. *Canadian Medical Association Journal*.

[24] Allen M. J., Asbridge M. M., Macdougall P. C., Furlan A. D., Tugalev O. (2013). Self-reported practices in opioid management of chronic noncancer pain: a survey of Canadian family physicians. *Pain Research and Management*.

