# Supplementary material for: Perceptions and Impact of the 2017 Canadian Guideline for Opioid Therapy and Chronic Noncancer Pain: A Cross-Sectional Study of Canadian Physicians
Source: Pain Res Manag. 2020 Feb 17;2020:8380171. doi: 10.1155/2020/8380171 (PMC7048936; doi:10.1155/2020/8380171)
Supplement: Supplementary Materials — Appendix: Opioid Guideline Survey. [file 8380171.f1.pdf]

Appendix 1: Opioid Guideline Survey

1. Do you currently prescribe opioids for chronic noncancer pain? By opioids, we mean both weak opioids (codeine, tramadol) and stronger opioids (morphine, fentanyl, etc.)

   - ☐ Yes
   - ☐ No (Termination message)

2. Select the specialty/sub-specialty that is most closely related to the <u>main</u> area of your current practice/residency.

- ☐ Adolescent Medicine
- ☐ Anatomical Pathology
- ☐ Anesthesiology
- ☐ Cardiac Surgery
- ☐ Cardiology - Adult
- ☐ Cardiology - Pediatric
- ☐ Child and Adolescent Psychiatry
- ☐ Clinical Immunology & Allergy - Adult
- ☐ Clinical Immunology & Allergy – Pediatric
- ☐ Clinical Pharmacology & Toxicology
- ☐ Colorectal Surgery
- ☐ Critical Care Medicine - Pediatric
- ☐ Critical Care Medicine – Adult
- ☐ Dermatology
- ☐ Developmental Pediatrics
- ☐ Diagnostic Radiology
- ☐ Emergency Medicine
- ☐ Endocrinology & Metabolism – Adult
- ☐ Endocrinology & Metabolism – Pediatric
- ☐ Family Medicine / General Practice
- ☐ Forensic Pathology
- ☐ Forensic Psychiatry
- ☐ Gastroenterology - Adult
- ☐ Gastroenterology - Pediatric
- ☐ General Internal Medicine
- ☐ General Pathology
- ☐ General Surgery
- ☐ General Surgical Oncology
- ☐ Geriatric Medicine
- ☐ Geriatric Psychiatry
- ☐ Gyn. Reproductive Endocrinology & Infertility
- ☐ Gynecologic Oncology
- ☐ Hematological Pathology
- ☐ Hematology
- ☐ Infectious Diseases – Adult
- ☐ Infectious Diseases – Pediatric
- ☐ Internal Medicine
- ☐ Maternal Fetal Medicine
- ☐ Medical Biochemistry
- ☐ Medical Genetics
- ☐ Medical Microbiology
- ☐ Medical Oncology
- ☐ Neonatal Perinatal Medicine
- ☐ Nephrology – Adult
- ☐ Nephrology – Pediatric
- ☐ Neurology - Adult
- ☐ Neurology - Pediatric
- ☐ Neuropathology
- ☐ Neuroradiology
- ☐ Neurosurgery

- ☐ Nuclear Medicine
- ☐ Obstetrics & Gynecology
- ☐ Occupational Medicine
- ☐ Ophthalmology
- ☐ Orthopedic Surgery
- ☐ Otolaryngology - Head and Neck Surgery
- ☐ Pain Medicine
- ☐ Palliative Medicine
- ☐ Pediatric Emergency Medicine
- ☐ Pediatric Hematology/Oncology
- ☐ Pediatric Radiology
- ☐ Pediatric Surgery
- ☐ Pediatrics - General
- ☐ Physical Medicine & Rehabilitation
- ☐ Plastic Surgery
- ☐ Psychiatry
- ☐ Public Health and Preventive Medicine
- ☐ Radiation Oncology
- ☐ Respirology – Adult
- ☐ Respirology – Pediatric
- ☐ Rheumatology – Adult
- ☐ Rheumatology – Pediatric
- ☐ Thoracic Surgery
- ☐ Urology
- ☐ Vascular Surgery

☐ Other area of focus, e.g., Addiction Medicine. Please specify. ______________________________

2a. Please specify your area of focus.

3. In what province / territory do you <u>primarily</u> work?

- ☐ Alberta
- ☐ British Columbia
- ☐ Manitoba
- ☐ New Brunswick
- ☐ Newfoundland and Labrador
- ☐ Northwest Territories
- ☐ Nova Scotia
- ☐ Nunavut
- ☐ Ontario
- ☐ Prince Edward Island
- ☐ Quebec
- ☐ Saskatchewan
- ☐ Yukon

4. What is your age group?

- ☐ < 25
- ☐ 25 - 34
- ☐ 35 – 44
- ☐ 45 - 54
- ☐ 55 - 64
- ☐ 65 +
- ☐ I prefer not to answer

5. With what gender do you identify?

- ☐ Woman
- ☐ Man
- ☐ I do not identify within the binary system. I identify as (please specify if you wish):
- ☐ I prefer not to specify

6. With respect to your <u>main</u> patient care setting, what <u>best</u> describes the population primarily served by you.

- ☐ Urban/suburban
- ☐ Small town
- ☐ Rural
- ☐ Geographically isolated/remote
- ☐ Cannot identify a primary geographic population

7. What proportion (%) of your patient population is currently seeking care for chronic noncancer pain?

☐ <6% ☐ 21-50%
☐ 6-10% ☐ 51 – 75%
☐ 11-20% ☐ >75%

8. To what proportion (%) of your chronic noncancer pain patients do you prescribe opioids?

☐ <6% ☐ 21-50%
☐ 6-10% ☐ 51 – 75%
☐ 11-20% ☐ >75%

9. Before you started this survey, were you aware of the 2017 Canadian Guideline for Opioids and Chronic Noncancer Pain (the "2017 Guideline")?

☐ Yes ☐ No (survey will go to Comments option, and then close)

10. Have you read the 2017 Guideline?

☐ Yes, thoroughly
☐ Yes, parts of it
☐ No, but I anticipate that I will (survey will go to Comments option, then close)
☐ No, and I don't anticipate that I will (survey will go to Comments option, then close)

11. Did you read the print version or the online MAGICapp version of the guideline?

☐ Print version only (goes to 11.a.i)
☐ Online MAGICapp version only (goes to 11.b.i)
☐ Both (goes to 11.a.i [11.a.ii.], then 11.b.i. [11.b.ii.], then 11.c.i)

11.a.i. How would you rate the format of the print version of the 2017 Guideline?

☐ Excellent
☐ Good
☐ Poor (goes to 11.a.ii)

- ☐ Very poor (goes to 11 a.ii)
- ☐ Unsure / Don’t remember

11.a.ii. Why do you feel the format was poor? (select all that apply)

- ☐ Impractical for use in clinical practice
- ☐ No patient decision-aids for recommendations
- ☐ Other (please provide details in the text box)_________________________

11.b.i. How would you rate the format of the online MAGICapp version of the 2017 Guideline?

- ☐ Excellent
- ☐ Good
- ☐ Poor (goes to 11.b.ii)
- ☐ Very poor (goes to 11.b.ii)
- ☐ Unsure / Don’t remember

11.b.ii Why do you feel the format was poor? (select all that apply)

- ☐ Impractical for use in clinical practice
- ☐ The multi-layered format is overly complex
- ☐ Other (please provide details in the text box)______________________

11.c.i. Which version do you prefer? (only for those that answered “Both” for Question 11)

- ☐ The print version
- ☐ The online MAGICapp version
- ☐ No preference between the 2 versions

12. Rate how understandable (or clear) the 2017 Guideline is.

☐ Excellent
☐ Good
☐ Poor
☐ Very poor
☐ Unsure

Please explain your response:_________________________________

13. In general, did you find the 2017 Guideline recommendations to be feasible to implement?

☐ Very feasible
☐ Somewhat feasible
☐ Not feasible (goes to 13.a.i)
☐ Unsure

13.a.i. Why did you feel the recommendations were not generally feasible?

☐ Recommendations were too **restrictive** regarding use of opioids for chronic noncancer pain
☐ Recommendations were too **permissive** regarding use of opioids for chronic noncancer pain
☐ Recommendations were impractical
☐ Recommendations were unclear
☐ Other (please provide details in the text box)_______________________

Please explain your response:____

14. Are there any areas where your current practice differs from what is recommended in the Guideline?

☐ Yes (answer next question)
☐ No (go to Question 16)

15. Have you made any changes in your treatment of patients with chronic noncancer pain because of the 2017 Guideline recommendations

☐ Yes – Please explain________________________

☐ No, but I plan to

☐ No, and I don't plan to

16. To what extent do you agree with the following statement?
The 2017 Guideline has important advantages over other related guidelines.

☐ Strongly agree
☐ Agree
☐ Uncertain [go straight to Q20]
☐ Disagree
☐ Strongly disagree
☐ Not applicable – there are no other guidelines in my jurisdiction [Skip to question 20]

17. Which other opioid and chronic pain guideline(s) have you consulted?

____

18. What advantages does the 2017 Guideline have over the others? (check all that apply) (only for Strongly Agree or Agree replies in question 16)

☐ national in scope
☐ broadly endorsed
☐ more specific guidance in certain areas
☐ better evidence review process
☐ other. Please specify____

19. What advantages do other guidelines have over the 2017 Guideline? (only for Disagree or Strongly Disagree replies in question 16)________________________

20. To what extent do you agree with the following statement?
The 2017 Guideline is evidence-based.

☐ Strongly agree ☐ Agree ☐ Uncertain ☐ Disagree ☐ Strongly disagree

21. Is it your understanding that the 2017 Guideline <u>mandates</u> that you taper all patients currently prescribed high-dose opioids for chronic noncancer pain to below 90mg morphine equivalent dose (MED) per day?

☐Yes ☐ Uncertain ☐No

22. Would you benefit from support to manage opioid tapering efforts for your patients currently prescribed high dose opioid therapy for chronic noncancer pain (≥90mg MED per day):

- ☐ Yes – What support would be helpful?___________________________________
- ☐ Uncertain
- ☐ No
- ☐ Not applicable - none of my patients are prescribed ≥90mg MED per day

23. What factors make it challenging for you as a physician to follow the 2017 Guideline? (Check all that apply)

- ☐ Inadequate time to deal with complex cases
- ☐ Patient reluctance to cooperate in the approaches recommended
- ☐ Need for **better** training in chronic pain management
- ☐ Need for **more** training in chronic pain management
- ☐ Lack of health insurance coverage for patients to non-pharmacologic treatment alternatives for chronic noncancer pain (e.g., physical therapy)
- ☐ Lack of existing services in community that offer non-pharmacologic treatment alternatives for chronic noncancer pain
- ☐ Lack of health insurance coverage for addiction treatment and management (e.g. methadone, Suboxone®)
- ☐ Lack of existing services in community for addiction treatment and management
- ☐ Need for better continuing education in in addiction
- ☐ Lack of access to specialists who can advise me
- ☐ Unrealistic to expect tapering of some patients currently prescribed high-dose opioids to or below 90mg MED per day
- ☐ The 2017 Guideline itself is unclear
- ☐ Other _______________________
- ☐ There are no challenges

24. What could help you implement the 2017 Guideline recommendations? (Check all that apply)

- ☐ Access to Continuing Medical Education on opioid prescribing
- ☐ Access to Continuing Medical Education on opioid de-prescribing
- ☐ Access to Continuing Medical Education on non-opiate medical pain management approaches
- ☐ Access to guideline implementation tools and supports, such as self-assessments, checklists, and algorithms

- ☐ Access to training on evidence based approaches to supporting behaviour change
- ☐ Real-time access to prescription monitoring database
- ☐ Clinical decision support system integrated within my EMR
- ☐ Mentorship programs in chronic noncancer pain and addiction that link me with more experienced colleagues
- ☐ More specialized chronic pain treatment services in my area
- ☐ More chronic pain treatment options, covered by health insurance
- ☐ More addiction treatment services in my area
- ☐ Billing incentives to increase time with complex chronic noncancer pain patients
- ☐ Other______________________
- ☐ I do not require any additional tools or assistance

25. If you could participate in just 3 CME/CPD programs on opioid prescribing in the coming year, what topic areas would you choose? Assume the format of the session is the one you prefer. (Select up to 3 options)

- ☐ Non-opioid options to pain management, pharmacologic and non-pharmacologic
- ☐ Screening, initiating and monitoring patients on opioid therapy.
- ☐ Managing the demanding, resistant or non-adherent patient on chronic opioids
- ☐ Safely tapering opioids
- ☐ Initiating opioid substitution therapy in my practice (methadone, buprenorphine / naloxone or Suboxone®)
- ☐ Preventing and managing opioid overdoses
- ☐ Community based programs to reduce the impact of opioid addictions and death
- ☐ Other: ____

26. What format of Continuing Medical Education would you find <u>most</u> helpful? Choose one

- ☐ Online learning: live interactive webinars
- ☐ Online learning: courses or YouTube style archived video
- ☐ Small group learning
- ☐ Mentoring with an expert colleague
- ☐ Practice audit: support in gathering and analyzing my own practice data
- ☐ Lectures
- ☐ Other – please specify______________________________

27. Since the launch of the 2017 Guideline, have you made any of the following changes? (check all that apply)

- ☐ Reduced the number of new starts of opioid medications compared to before (finish, to last opportunity for comment)
- ☐ Prescribed opioids to more patients with chronic noncancer pain (finish, to last opportunity for comment)
- ☐ Decided not to prescribe opioids for patients with chronic noncancer pain who also have an active substance use disorder (go to next question)
- ☐ Decided not to prescribe opioids for patients with chronic noncancer pain who also have an active psychiatric disorder, aside from substance use disorder (go to next question)
- ☐ Decided not to prescribe opioids for specific patients with chronic noncancer pain based on criteria other than having an active substance use disorder or an active psychiatric disorder (go to next question)
- ☐ Decided not to prescribe opioids for any patients with chronic noncancer pain (go to next question)
- ☐ Reduced the dosage of opioids I prescribe for chronic noncancer pain (new starts) compared to before (finish, to last opportunity for comment)
- ☐ Tapered the dose of opioids I prescribe to some existing patients (finish, to last opportunity for comment)
- ☐ Reduced the number of pills dispensed at one time in opioid prescriptions that I write (finish, to last opportunity for comment)
- ☐ Other changes – please specify______________________________ (finish, to last opportunity for comment)
- ☐ No changes (finish, to last opportunity for comment)

28. Please explain why you have chosen not to prescribe opioids for chronic noncancer pain to certain populations, or at all?

____

Thank you for taking the time to share your views with us. If you have any questions about this survey, please send an email to yourvoice@cma.ca.
